# Usefulness of Herpes Consensus PCR methodology to routine diagnostic testing for herpesviruses infections in clinical specimens

**DOI:** 10.1186/1743-422X-4-59

**Published:** 2007-06-12

**Authors:** Georgia Vrioni, Christos Kalogeropoulos, Constantina Gartzonika, Efthalia Priavali, Stamatina Levidiotou

**Affiliations:** 1 Department of Microbiology, Medical School, University of Ioannina, Greece; 2 Department of Ophthalmology, University Eye Clinic of Ioannina, Greece

## Abstract

The purposes of the study were to assess the usefulness of simultaneously amplifying herpes simplex virus 1 and 2, varicella-zoster virus, cytomegalovirus, Epstein-Barr virus and human herpesvirus 6 DNA in various clinical specimens and to analyze clinical events in patients presenting positive results. A total of 763 clinical samples obtained from 758 patients, including 115 cerebrospinal fluids, 102 aqueous fluids, 445 swabs from genital (152), oro-facial (138) and other (155) skin lesions, 96 eye swabs and 5 bronchoalveolar lavages, were tested by using the Consensus polymerase chain reaction methodology. The clinical files of the patients were consulted retrospectively. 171 of the 758 patients (22.5%) were positive for at least one of the six target viruses: herpes simplex virus 1 (n = 95), varicella-zoster virus (n = 40), herpes simplex virus 2 (n = 21), herpes simplex virus 1 plus herpes simplex virus 2 (n = 8), cytomegalovirus (n = 4), Epstein-Barr virus (n = 1), human herpesvirus 6 (n = 1), and herpes simplex virus 1 plus human herpesvirus 6 (n = 1). The Consensus methodology enabled the rapid and accurate detection of herpesviruses in various clinical specimens and provided a reliable tool in the diagnosis of herpetic infections.

## Findings

Molecular assays based on PCR have become an important tool for the detection of various herpes virus DNA in clinical specimens [1-5]. Since clinical features of herpetic infections are often not specific and cannot distinguish between the different viruses, amplification systems that allow simultaneous detection of different herpesviruses, have become available [6-8]. The Herpes Consensus PCR methodology (Argene Biosoft, Varilhes, France) uses "stair primers" [9] in which the primer sequences are based on consensus sequences in the DNA polymerase gene of herpesviruses and thus allows the simultaneous detection of the six most frequently encountered viruses – herpes simplex virus 1 and 2 (HSV-1, HSV-2), varicella-zoster virus (VZV), cytomegalovirus (CMV), Epstein-Barr virus (EBV) and human herpesvirus 6 (HHV-6)- by a single PCR [10].

The purpose of this study was to report our experience over the last four years in the detection of the six most common herpesviruses in various clinical specimens by using Consensus PCR methodology and to investigate the correlation between the results obtained with this technique and clinical data.

A total of 763 specimens [115 cerebrospinal fluids (CSF), 102 aqueous fluids, 152 genital swabs, 155 skin swabs, 138 oro-facial swabs, 96 eye swabs and 5 bronchoalveolar lavages (BAL)] from 758 patients with a range of clinical presentations that included central nervous system (CNS) herpesvirus-associated clinical signs such us fever with acute behavioral disturbances, seizures and focal neurological signs; keratitis or/and conjunctivitis, uveitis (presented in the majority of cases as iridocyclitis) and retinitis, regarding ocular manifestations; genital, oral, or skin lesions and pneumonitis. All CSF samples were tested for biochemical analysis (the concentration of protein and glucose and lymphocyte count) and bacterial infection (by culturing). Moreover, CSF samples showed normal glucose level and proved negative when cultured for bacterial infections. The prospective study reported here was carried on specimens received in Microbiology Department of University Hospital of Ioannina (a central reference hospital in northwestern Greece) between March 2002 and March 2006 on a daily basis from hospital in- and outpatients as well as from those being served by general practitioners and doctors in specialized infectious-disease clinics. The study underwent ethics review and approval.

PCR was performed according to the protocol of the commercial Herpes Consensus Generic test (Argene Biosoft, Varilhes, France), which uses gene amplification to search for the six main human herpes viruses implicated in infections, HSV-1, HSV-2, CMV, EBV, VZV and HHV-6. The sequences of the six viruses were amplified simultaneously in a single "monotest" tube, using the stair primers technique following the manufacturer's recommendations. These specific primers consisted of sequences correspond to a conserved region of the DNA polymerase gene and allowed efficient amplification of the 3' mutated sequences [10]. Positive controls, included in the kit, corresponding to plasmids, containing respectively the six herpesvirus sequences recognized by the primers, were used as amplification positive samples. The absence of inhibitors was demonstrated through the use of a sample inhibition premix provided in the kit. Positive samples were further analyzed through Hybridowell Herpes Identification test (Argene Biosoft, Varilhes, France) with six Herpesvirus specific biotinylated probes in order to determine the exact virus involved. The test required about seven hours for processing nucleic acid extraction (one hour), amplification (three hours) and detection (three hours) and three more hours for identification, when the Consensus Generic test's result was positive. The manufacturer guarantees the sensitivity of the kit to be 100 genomes per PCR. The analytical sensitivity and specificity were determined with Quality Control for Molecular Diagnostics (QCMD) Varicella zoster virus proficiency panel 2003 consisted of 12 coded samples: nine samples of a dilution series of VZV and HSV-1, and two samples with no virus. Serial dilutions of positive controls, included in the kit, were also tested. To analyze for clinical specificity of the PCR assay, we studied 50 CSF samples from patients with chronic neurological disorders who were clinically not suspected to have viral CNS infection. The clinical files of the patients were consulted retrospectively.

Herpesvirus DNA was detected in 176 of 763 specimens collected from 171 patients (five patients tested positive in both BAL and skin swab specimens) (Table 1). Of these, 38 specimens were from children (38/176, 21.6%), whereas 135 (135/763, 17.7%) of the specimens studied were collected from equal number of children (54 CSF, 22 skin swabs, 47 oro-facial swabs and 12 eye swabs).

**Table 1:** Global results of specimen types and numbers positive for herpesviruses

Specimen type	No. tested	No. positive							
		
		HSV-1	HSV-2	CMV	EBV	VZV	HHV-6	HSV-1 plus HSV-2	HSV-1 plus HHV-6
CSF	115	2	-	4	1	1	1	-	1
Aqueous fluids	102	5	-	-	-	3	-	-	-
Genital swabs	152	9	21	-	-	-	-	8	-
Skin swabs	155	48	-	-	-	36^a^	-	-	-
Oro-facial swabs	138	15	-	-	-	-	-	-	-
Eye swabs	96	16	-	-	-	-	-	-	-
BAL	5	-	-	-	-	5^a^	-	-	-

Total	763	95	21	4	1	45	1	8	1

HSV-1, herpes simplex virus 1; HSV-2, herpes simplex virus 2; VZV, varicella-zoster virus; CMV, cytomegalovirus; EBV, Epstein-Barr virus; HHV-6, human herpesvirus 6; BAL, bronchoalveolar lavage.

^a ^BAL specimens were from five patients with five VZV positive skin swabs.

Ten out of 115 patients tested positive in CSF specimen. HSV-1 was detected in 2 adults with suggestive clinical signs of encephalitis. Computerized tomography scan, electroencephalogram and magnetic resonance imaging supported herpesvirus encephalitis. CMV was detected in 2 children with clinical signs of meningoencephalitis and 2 adults, one with non-Hodgkin lymphoma and the other with chronic lymphocytic leukemia, both in chemotherapy, and signs precented encephalitis. Moreover, plasma specimens tested from these patients with a commercial quantitative CMV PCR (Roche Diagnostic Systems) were positive with more than 400 CMV DNA copies per ml (data not shown). VZV was detected in one child presented fever, headache and neck stiffness six days after the appearance of rash. EBV and HHV-6 were detected in HIV-positive adult patients with encephalitis. HSV-1 and HHV-6 co-infection was detected in one immunocompetent patient with encephalitis. All patients had abnormal CSF leukocyte counts (range, 120–580/mm^3^) and high CSF protein levels. All patients with HSV and VZV CNS infections improved upon acyclovir (ACV) therapy. Analysis of CSF samples obtained after completion of the antiviral treatment gave negative results. Patients with CMV infection improved upon ganciclovir therapy, except the one with chronic leukemia who died. The HIV-positive patients were referred in another hospital and so no other clinical information was available.

The far majority of specimens (541/763, 70.9%) were swabs from ano-genital, skin, oro-facial and eye vesicles or lesions suspected to be due to HSV or VZV infection. HSV-1 was detected in 9 adults with genital vesicular lesions, 48 with orolabial lesions, 15 children with gingivostomatitis, 8 adults with keratoconjunctivitis and 8 children with unilateral conjunctivitis and herpes labialis. HSV-2 and HSV-1/HSV-2 co-infection were detected in 29 patients with ano-genital lesions. All these patients were recovered upon ACV therapy, except 2 children with gingivostomatitis and 6 patients with genital herpes who improved spontaneously. VZV was detected in 12 children with rash and in 24 adults with herpes zoster. Five adults with herpes zoster developed pneumonia 5 to 6 days after the onset of rash and VZV was also detected in BAL. Patients with herpes zoster were improved upon valaciclovir (7 patients) or famciclovir (12 patients) therapy, and those with varicella pneumonitis upon ACV therapy.

Eight out of 102 patients detected positive in aqueous fluid: HSV-1 was detected in 5 and VZV in 2 patients with uveitis and one patient with retinitis. The patients with HSV-1 infection experienced in their majority (4 out of 5) hypertensive iridocyclitis (increase of intraocular pressure and anterior uvea inflammation) along with iris alterations (ie: sectoral atrophy); only in one HSV-1 positive patient the examination of the fundus of the eye revealed posterior uveitis and inflammatory changes of retinal vessels (vasculitis). On the other hand VZV intraocular infection presented in 2 patients as hypertensive iridocyclitis and in one patient as acute retinal necrosis syndrome, a very severe form of viral retinitis characterized by full-thickness retinal necrosis and vasculitis along with iridocyclitis and vitritis. ACV was administered systemically in all patients and in cases with iridocyclitis topically as well. Inflammation was controlled in all cases during the first week and regressed afterwards resulting in satisfactory or even excellent visual outcome. However, in one case (acute retinal necrosis syndrome) intraocular surgery was needed besides in order to secure the anatomic integrity of the eye with good results.

Positive results obtained in Consensus assay were confirmed by targeted PCR (data not shown) [11]. Results from the QCMD VZV proficiency panel and controls' dilutions were identical to the expected ones and none of the CSF specimens from patients clinically not suspected of having herpesviruses CNS infections were positive with the Consensus PCR assay.

To our knowledge, this is the first time that the experience of a single clinical microbiology laboratory has been reported since the commercial availability of the improved Herpes Consensus PCR assay in Greece. The assay's ability to detect herpesviruses in a wide range of specimen types in a single amplification enhances its utility in the clinical setting, by avoiding the need to test samples separately for each virus and reducing the time (8 hours for processing amplification and detection) and costs of the tests. The Consensus PCR assay enabled detection of viruses which were not requested by the clinicians in several cases, showing that the same clinical syndrome may be produced by different herpesviruses [10,12-14]. Additionally, as reported by other authors [10,13,15,16], the assay made it possible to detect and identify some cases of mixed herpetic infections that would otherwise probably have gone unobserved. The significance of these coinfections is not clear; however their recognition is important to ensure adequate antiviral treatment when available. The ability of Consensus PCR to yield results from ocular specimens has been of particular interest and of considerable clinical benefit [13,15], especially in cases of hypertensive iridocyclitis without concurrent corneal manifestations [17-19]. Finally, negative results after antiviral therapy suggest that the Consensus PCR assay may also be useful for monitoring the efficacy of treatment [20].

According to our experience, Consensus PCR assay can be useful to facilitate the routine diagnosis of herpesviruses infections, within a single assay, single clinical sample, thereby allowing earlier application of specific antiviral treatment and better clinical management.

## Competing interests

The author(s) declare that they have no competing interests.

## Authors' contributions

GV conceived of the study, participated in its design, carried out the PCR method and drafted the manuscript. CG and EP carried out the PCR method and analyzed the results. CK participated in the design of the study, collected the aqueous fluid and analyzed the results from patients with ocular manifestations. SL participated in the design of the study and helped to draft the manuscript. All authors read and approved the final manuscript
